# Supplementary figures and images for: Gas chromatography-mass spectrometry and Fourier-transform infrared spectroscopy coupled to chemometrics for metabolome analysis of different milk types in the light of green analytical chemistry
Source: PeerJ. 2025 Sep 17;13:e19921. doi: 10.7717/peerj.19921 (PMC12449862; doi:10.7717/peerj.19921)

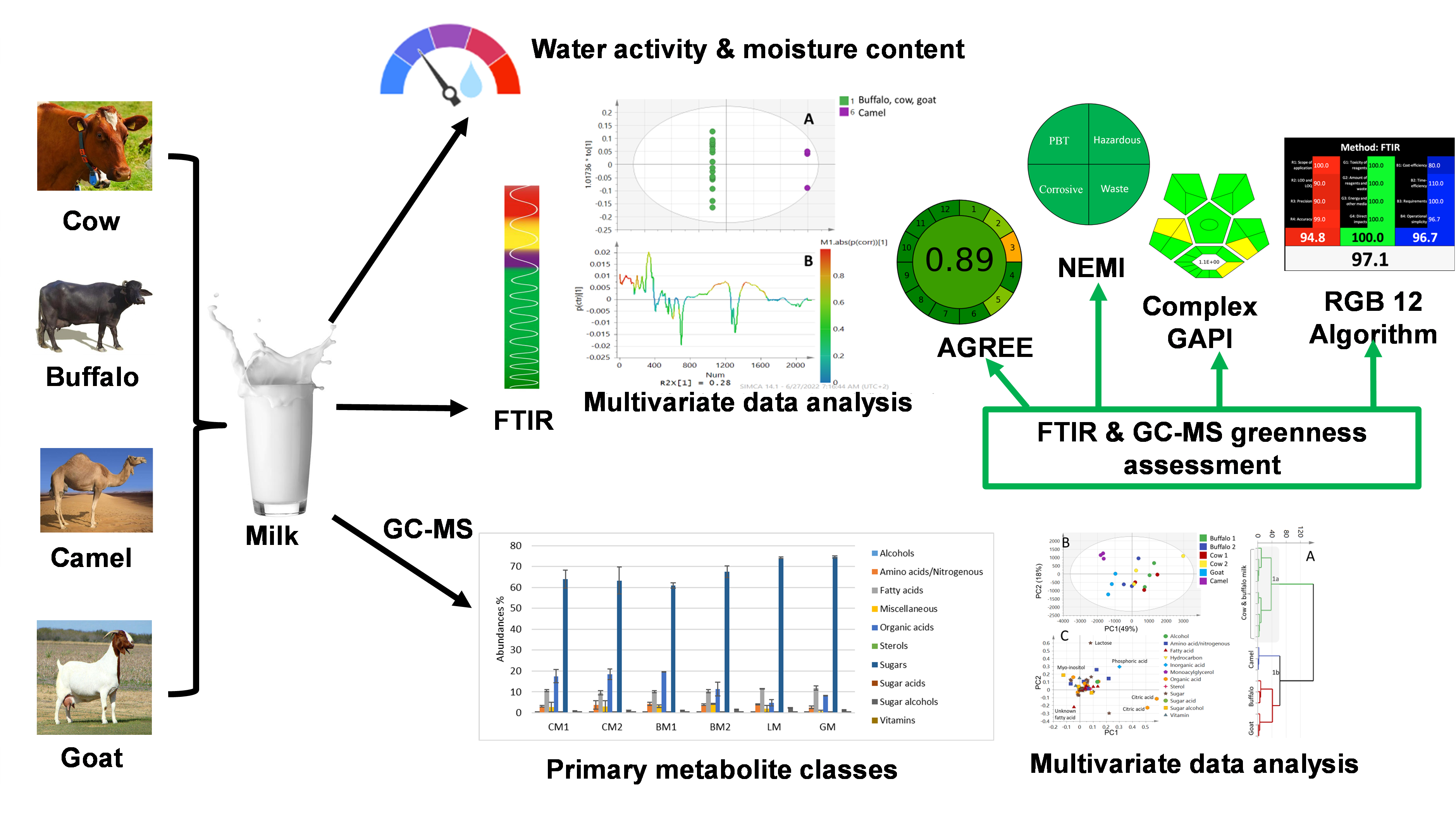

Supplement: Supplemental Information 1 [file peerj-13-19921-s001.png]

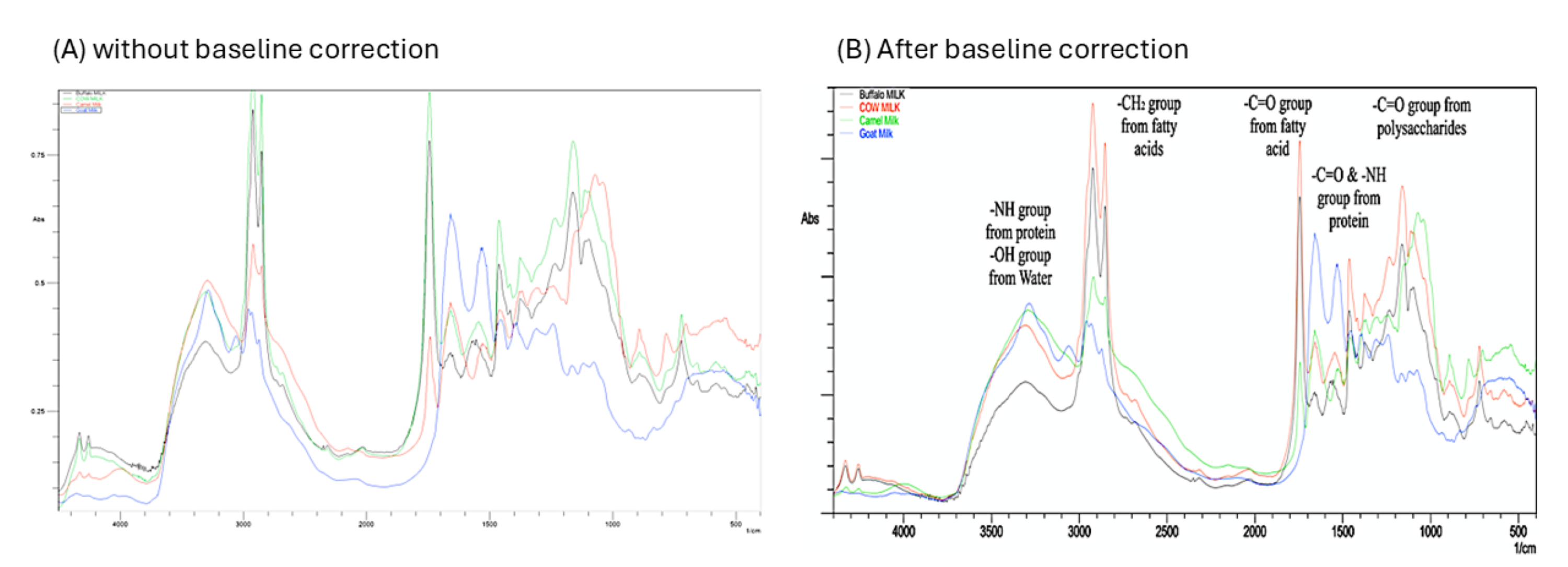

Supplement: Supplemental Information 5 [file peerj-13-19921-s005.png]

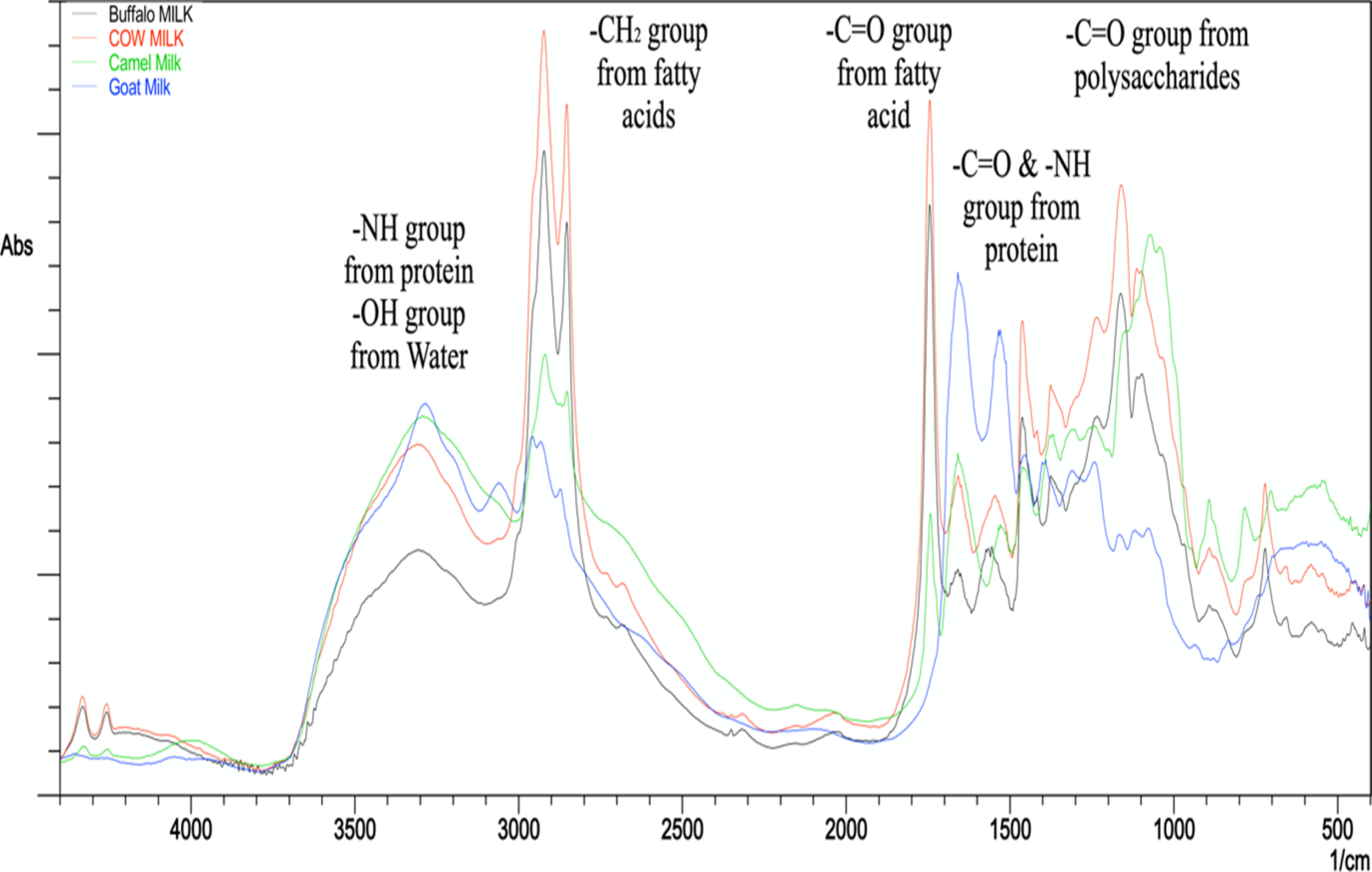

Supplement: Supplemental Information 6 [file peerj-13-19921-s006.png]

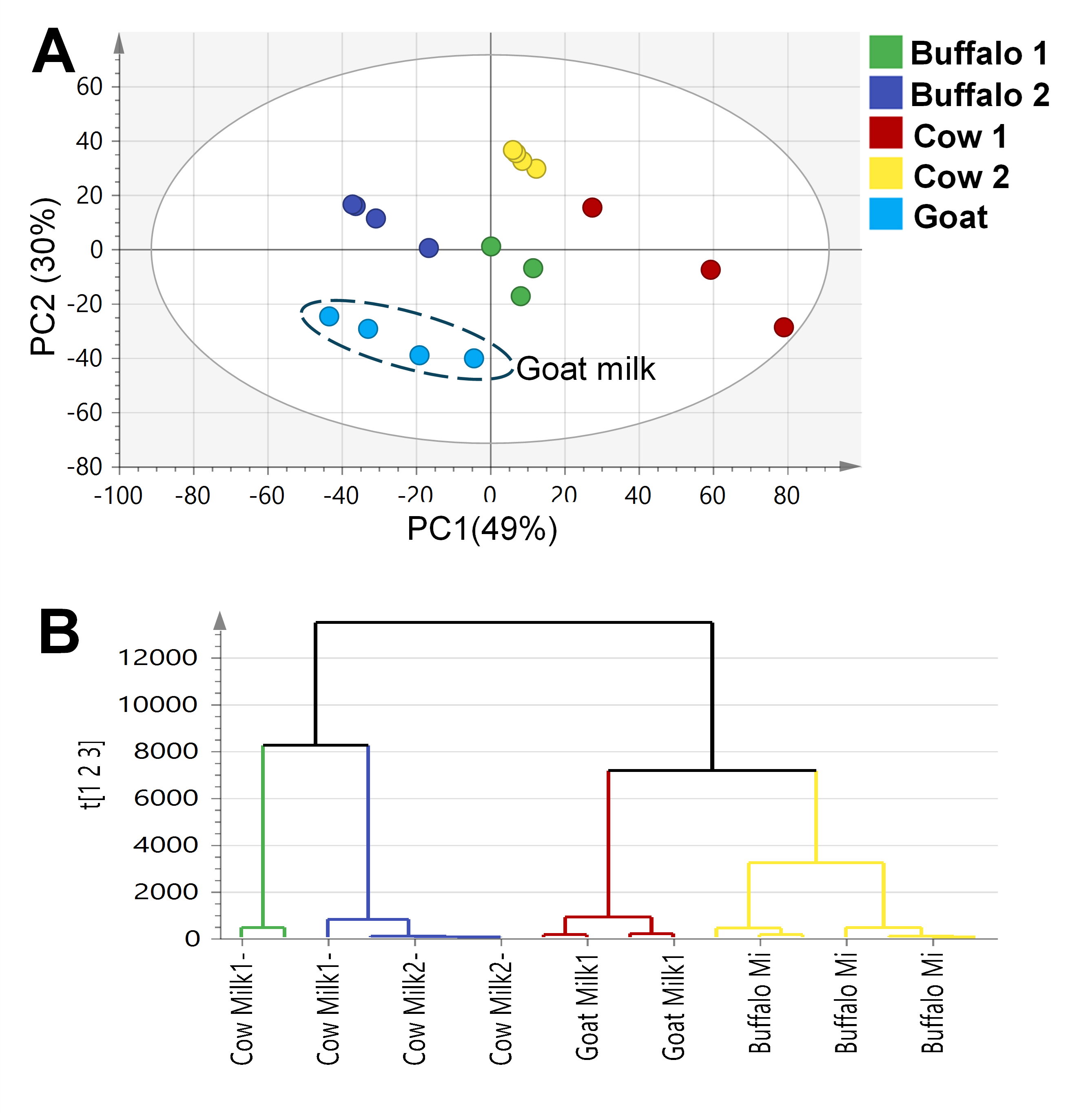

Supplement: Supplemental Information 7 [file peerj-13-19921-s007.png]

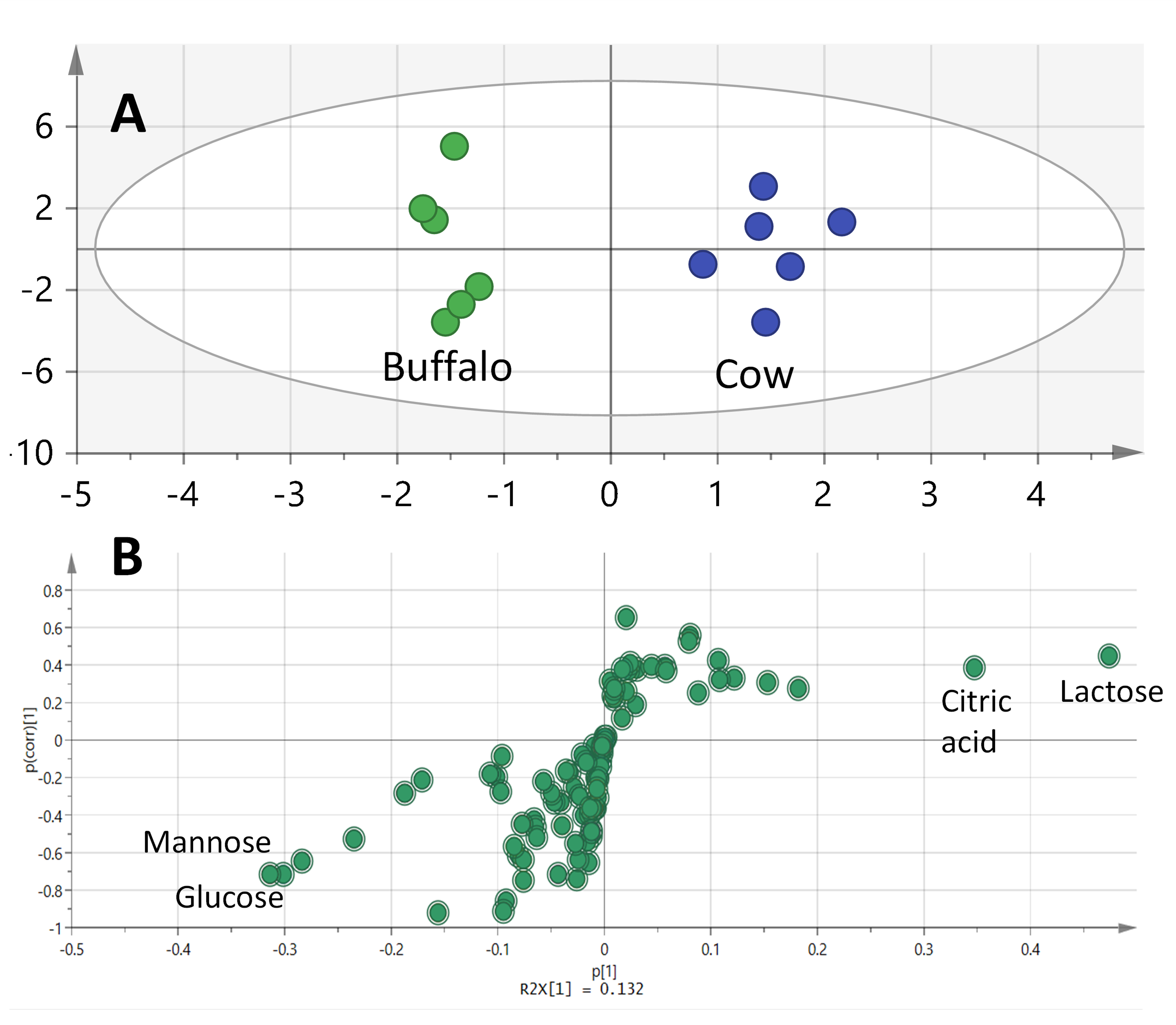

Supplement: Supplemental Information 8 — GC-MS-based OPLS-DA score plot derived from modelling cow against buffalo milk (A). The respective S-plot (B) shows the covariance p[1] against the correlation p(cor)[1] of the variables of the discriminating component of the OPLS-DA model. Cut-off values of P < 0.05 were used; selected variables are highlighted in the S-plot with identifications. [file peerj-13-19921-s008.png]
