# Supplementary material for: Gas chromatography-mass spectrometry and Fourier-transform infrared spectroscopy coupled to chemometrics for metabolome analysis of different milk types in the light of green analytical chemistry
Source: PeerJ. 2025 Sep 17;13:e19921. doi: 10.7717/peerj.19921 (PMC12449862; doi:10.7717/peerj.19921)
Supplement: Supplemental Information 2 [file peerj-13-19921-s002.docx]

**Table S1: Milk specimens codes:**

| **Samples names** | **Animals names/ breeds** | **Samples codes** | **Geographical source** |
| --- | --- | --- | --- |
| - Cow milk from different sources. | Baladi cattle  Holstein Friesian  *Bos taurus* | CM1, CM2, CM3, CM4, CM5, CM6, CM7, CM8 | Elwahat Albahareya, Giza, Egypt |
| - Buffalo milk from different sources. | River buffalo  *Bubalus bubalis* | BM1, BM2, BM3, BM4, BM5, BM6, BM7, BM8 |  |
| - Camel milk from different sources. | Arabian camel  *Camelus dromedarius* | LM1, LM2, LM3, LM4, LM5, LM6, LM7, LM8 |  |
| - Goat milk from different sources. | Zaraibi goat  *Capra aegagrus hircus* | GM1, GM2, GM3, GM4, GM5, GM6, GM7, GM8 |  |
