# Supplementary material for: Gas chromatography-mass spectrometry and Fourier-transform infrared spectroscopy coupled to chemometrics for metabolome analysis of different milk types in the light of green analytical chemistry
Source: PeerJ. 2025 Sep 17;13:e19921. doi: 10.7717/peerj.19921 (PMC12449862; doi:10.7717/peerj.19921)
Supplement: Supplemental Information 3 [file peerj-13-19921-s003.docx]

**Table S2: Water activity and moisture content of milk samples:**

| **Milk sample** | **Water activity (a_w_)** | **Average Moisture content (MC%)** |
| --- | --- | --- |
| CM | 0.9944 | 87.31 |
| BM | 0.9955 | 88.95 |
| LM | 0.9936 | 88.86 |
| GM | 0.9962 | 87.99 |
