# Supplementary material for: Gas chromatography-mass spectrometry and Fourier-transform infrared spectroscopy coupled to chemometrics for metabolome analysis of different milk types in the light of green analytical chemistry
Source: PeerJ. 2025 Sep 17;13:e19921. doi: 10.7717/peerj.19921 (PMC12449862; doi:10.7717/peerj.19921)
Supplement: Supplemental Information 4 [file peerj-13-19921-s004.docx]

**Table S3: Primary metabolites analysis using GC-MS from different milk types. Results are expressed as a relative percentile (n=3) of the total peak areas.** **Metabolites denoted by an asterisk represent those confirmed by the standard.**

| **KI** | **Name** | **Class** | **BM1** | **BM2** | **CM1** | **CM2** | **GM** | **LM** |
| --- | --- | --- | --- | --- | --- | --- | --- | --- |
|  |  |  | **Avg ± SD** | **Avg ± SD** | **Avg ± SD** | **Avg ± SD** | **Avg ± SD** | **Avg ± SD** |
| 1157 | Benzyl alcohol, TMS | Alcohol | 0.004±0.0002 | 0.004±0.001 | 0.003±0.002 | 0.003±0.002 | 0.004±0.002 | 0.003±0.001 |
| 1285 | Glycerol, tri TMS* |  | 0.53±0.22 | 0.35±0.23 | 0.33±0.23 | 0.56±0.31 | 0.16±0.02 | 0.23±0.03 |
| 1957 | 1-hexadecanol-O-TMS |  | 0.01±0.002 | 0.004±0.002 | 0.005±0.003 | 0.004±0.003 | 0.01±0.005 | 0.01±0.003 |
|  | **Total alcohols** | | **0.54 ± 0.23** | **0.36±0.23** | **0.34±0.23** | **0.56±0.31** | **0.17±0.03** | **0.23±0.03** |
| 1081 | Valine, TMS ester | Amino acid/ nitrogenous | 0.01±0.002 | 0.01±0.001 | 0.01±0.002 | 0.01±0.002 | 0.01±0.001 | 0.01±0.001 |
| 1141 | N-acetyl glycine TMS |  | 0.13±0.05 | 0.11±0.02 | 0.09±0.02 | 0.11±0.06 | 0.09±0.01 | 0.08±0.02 |
| 1149 | L-Pyroglutamic acid, methyl ester |  | 0.01±0.001 | 0.01±0.001 | 0.01±0.002 | 0.01±0.001 | 0.01±0.002 | 0.01±0.002 |
| 1160 | L-Leucine, TMS ester |  | 0.01±0.001 | 0.01±0.001 | 0.005±0.002 | 0.005±0.001 | 0.01±0.0004 | 0.01±0.001 |
| 1179 | Valine, di TMS |  | 1.27±0.65 | 1.05±0.33 | 0.80±0.39 | 1.18±1.22 | 0.70±0.13 | 0.45±0.17 |
| 1240 | Ethanol amine, N, N-di TMS-, TMS ether |  | 0.67±0.11 | 0.64±0.09 | 0.60±0.12 | 0.62±0.07 | 0.72±0.10 | 0.80±0.06 |
| 1250 | Urea, di TMS |  | 1.71±0.01 | 1.53±0.05 | 1.32±0.17 | 1.54±0.61 | 0.87±0.25 | 2.15±0.22 |
| 1275 | Ethanol amine, N, N-di TMS-, TMS ether |  | 0.03±0.002 | 0.12±0.02 | 0.03±0.02 | 0.02±0.02 | 0.11±0.03 | 0.32±0.03 |
| 1316 | Glycine, tri TMS* |  | 0.18±0.005 | 0.19±0.02 | 0.06±0.04 | 0.04±0.03 | 0.02±0.01 | 0.12±0.02 |
| 1431 | Aspartic acid, di TMS ester |  | 0.004±0.001 | 0.002±0.0001 | 0.001±0.0001 | 0.001±0.0004 | 0.001±0.0001 | 0.001±0.0001 |
| 1485 | Aspartic acid, N-TMS-, di TMS ester |  | 0.01±0.002 | 0.01±0.001 | 0.005±0.0004 | 0.01±0.002 | 0.005±0.0004 | 0.01±0.002 |
| 1537 | Pyroglutamic acid, di TMS |  | 0.08±0.002 | 0.05±0.01 | 0.02±0.01 | 0.02±0.01 | 0.01±0.004 | 0.03±0.01 |
| 1628 | Glutamic acid, tri TMS |  | 0.02±0.004 | 0.01±0.004 | 0.004±0.003 | 0.002±0.0003 | 0.001±0.0003 | 0.01±0.01 |
| 1758 | Orotic acid, tri TMS |  | 0.05±0.01 | 0.08±0.01 | 0.10±0.03 | 0.15±0.08 | 0.04±0.01 | 0.02±0.001 |
| 2101 | N-acetyl-D-glucosamine, tetra TMS ether |  | 0.02±0.004 | 0.02±0.01 | 0.05±0.04 | 0.11±0.09 | 0.02±0.005 | 0.01±0.002 |
| 2187 | L-Norvaline, N-(2-methoxyethoxycarbonyl)-, heptyl ester |  | 0.01±0.001 | 0.01±0.001 | 0.003±0.001 | 0.005±0.002 | 0.002±0.0004 | 0.002±0.001 |
|  | **Total amino acids/ Nitrogenous** | | **4.21±0.82** | **3.83±0.36** | **3.09±0.38** | **3.82±2.02** | **2.62±0.56** | **4.04±0.15** |
| 1460 | Capric acid, TMS ester | Fatty acid | 0.01±0.001 | 0.004±0.0002 | 0.003±0.002 | 0.01±0.003 | 0.003±0.0003 | 0.002±0.0002 |
| 1653 | Lauric acid, TMS ester |  | 0.01±0.001 | 0.01±0.001 | 0.01±0.01 | 0.01±0.01 | 0.01±0.0003 | 0.004±0.001 |
| 1848 | Myristic acid, TMS ester* |  | 0.05±0.002 | 0.02±0.005 | 0.02±0.01 | 0.03±0.02 | 0.01±0.002 | 0.01±0.002 |
| 1946 | n-Pentadecanoic acid TMS ester |  | 0.01±0.0001 | 0.01±0.002 | 0.01±0.002 | 0.01±0.005 | 0.004±0.0001 | 0.004±0.001 |
| 2044 | Palmitic acid, TMS* |  | 0.63±0.06 | 0.49±0.17 | 0.84±0.56 | 0.54±0.30 | 0.35±0.03 | 0.40±0.15 |
| 2145 | Margaric acid, TMS ester |  | 0.004±0.003 | 0.01±0.003 | 0.004±0.003 | 0.01±0.003 | 0.01±0.001 | 0.01±0.001 |
| 2216 | Oleic acid, TMS ester* |  | 0.24±0.02 | 0.12±0.02 | 0.12±0.07 | 0.15±0.11 | 0.10±0.01 | 0.19±0.13 |
| 2241 | Stearic acid, TMS ester* |  | 0.44±0.001 | 0.39±0.09 | 0.72±0.56 | 0.43±0.17 | 0.35±0.04 | 0.38±0.08 |
| 2396 | Myristic acid, 2,3-di-TMS-propyl ester |  | 0.02±0.002 | 0.01±0.003 | 0.005±0.002 | 0.004±0.002 | 0.002±0.001 | 0.003±0.001 |
| 2783 | Stearic acid, 2,3-di TMS propyl ester |  | 0.13±0.04 | 0.09±0.01 | 0.12±0.09 | 0.07±0.01 | 0.07±0.02 | 0.09±0.03 |
| 2799 | Unknown fatty acid |  | 8.53±0.56 | 9.21±0.88 | 8.74±0.76 | 8.36±1.60 | 10.97±1.06 | 10.51±0.34 |
|  | **Total fatty acids** | | **10.08±0.61** | **10.35±0.77** | **10.59±0.54** | **9.62±1.09** | **11.88±0.96** | **11.60±0.12** |
| 1282 | Unknown hydrocarbon | Hydrocarbon | 0.01±0.01 | 0.005±0.003 | 0.01±0.02 | 0.01±0.005 | 0.01±0.001 | 0.01±0.003 |
| 1288 | Phosphoric acid, tri TMS* | Inorganic acid | 3.03±0.64 | 4.25±0.33 | 2.55±2.21 | 2.93±2.69 | 0.63±0.63 | 1.86±1.60 |
| 2597 | 1-Monopalmitin TMS ether | Monoacylglycerol | 0.13±0.03 | 0.06±0.01 | 0.10±0.09 | 0.05±0.01 | 0.05±0.02 | 0.06±0.01 |
|  | **Total miscellaneous** | | **3.16±0.68** | **4.31±0.34** | **2.66±2.26** | **2.99±2.70** | **0.69±0.64** | **1.92±1.59** |
| 1077 | Glycolic acid, TMS ester | Organic acid | 0.02±0.01 | 0.01±0.003 | 0.01±0.002 | 0.01±0.002 | 0.01±0.002 | 0.01±0.003 |
| 1095 | Pyruvic acid, enol di TMS |  | 0.12±0.05 | 0.04±0.01 | 0.06±0.01 | 0.09±0.04 | 0.01±0.004 | 0.08±0.01 |
| 1112 | Oxalic acid, di TMS ester |  | 1.47±0.71 | 1.03±0.46 | 0.72±0.42 | 0.82±0.16 | 0.69±0.38 | 0.63±0.41 |
| 1139 | Methyl malonic acid, di TMS |  | 0.18±0.08 | 0.13±0.06 | 0.11±0.05 | 0.11±0.05 | 0.10±0.04 | 0.08±0.04 |
| 1146 | Oxalic acid, di TMS ester |  | 0.01±0.002 | 0.01±0.001 | 0.01±0.001 | 0.01±0.001 | 0.01±0.001 | 0.01±0.001 |
| 1167 | 3-Hydroxy butanoic acid, di TMS |  | 0.05±0.01 | 0.02±0.002 | 0.05±0.05 | 0.10±0.09 | 0.02±0.002 | 0.004±0.002 |
| 1224 | Succinic acid, di TMS |  | 0.18±0.06 | 0.15±0.02 | 0.12±0.02 | 0.15±0.08 | 0.14±0.01 | 0.13±0.02 |
| 1269 | Caprylic acid TMS ester |  | 0.01±0.002 | 0.01±0.001 | 0.01±0.001 | 0.01±0.002 | 0.01±0.0002 | 0.01±0.001 |
| 1321 | Succinic acid, di TMS |  | 0.09±0.01 | 0.07±0.005 | 0.07±0.05 | 0.13±0.12 | 0.08±0.01 | 0.65±0.07 |
| 1342 | Glyceric acid, tri TMS |  | 0.02±0.001 | 0.06±0.002 | 0.02±0.01 | 0.03±0.02 | 0.01±0.001 | 0.003±0.001 |
| 1352 | Fumaric acid, di TMS |  | 0.004±0.001 | 0.01±0.001 | 0.004±0.001 | 0.004±0.001 | 0.01±0.003 | 0.003±0.0002 |
| 1361 | Nonanoic acid, TMS ester |  | 0.77±0.03 | 0.78±0.03 | 0.74±0.04 | 0.77±0.01 | 0.92±0.03 | 0.97±0.05 |
| 1409 | Methyl succinic acid, di TMS |  | 0.01±0.002 | 0.01±0.001 | 0.01±0.002 | 0.01±0.002 | 0.01±0.001 | 0.01±0.001 |
| 1500 | Malic acid, tri TMS |  | 0.02±0.002 | 0.09±0.01 | 0.02±0.004 | 0.01±0.004 | 0.13±0.01 | 0.01±0.002 |
| 1583 | 2-Hydroxyglutaric acid, tri TMS |  | 0.02±0.001 | 0.01±0.001 | 0.02±0.01 | 0.04±0.03 | 0.01±0.001 | 0.01±0.0005 |
| 1614 | 2-ketoglutaric acid, tri TMS |  | 0.08±0.01 | 0.06±0.004 | 0.05±0.02 | 0.08±0.05 | 0.08±0.01 | 0.03±0.003 |
| 1757 | Cis-Aconitic acid, tri TMS |  | 0.01±0.0003 | 0.01±0.0001 | 0.01±0.003 | 0.01±0.01 | 0.004±0.0004 | 0.003±0.003 |
| 1836 | Citric acid, tetra TMS |  | 8.24±0.42 | 6.22±0.09 | 7.73±1.26 | 8.01±1.06 | 5.81±0.31 | 1.56±0.06 |
| 1841 | Citric acid, tetra TMS isomer |  | 8.24±0.42 | 2.65±3.09 | 7.74±1.26 | 8.02±1.06 | 0.16±0.02 | 0.57±0.91 |
|  | **Total organic acids** | | **19.52±0.12** | **11.35±3.20** | **17.49±3.12** | **18.41±2.59** | **8.21±0.11** | **4.77±1.41** |
| 3071 | Cholesterol, TMS* | Sterol | 0.15±0.06 | 0.09±0.04 | 0.80±1.25 | 0.10±0.05 | 0.07±0.02 | 0.09±0.04 |
|  | **Total sterols** | | **0.15±0.06** | **0.09±0.04** | **0.80±1.25** | **0.10±0.05** | **0.07±0.02** | **0.09±0.04** |
| 1271 | Glycolaldehyde dimer, di TMS ether | Sugar | 0.01±0.0003 | 0.01±0.003 | 0.01±0.003 | 0.01±0.002 | 0.01±0.004 | 0.01±0.001 |
| 1491 | Threose, tri TMS ether methyl oxime |  | 0.01±0.0002 | 0.01±0.001 | 0.005±0.002 | 0.01±0.003 | 0.01±0.0003 | 0.02±0.001 |
| 1633 | Arabinose, 2,3,4,5-tetra-O-TMS |  | 0.01±0.003 | 0.01±0.003 | 0.01±0.01 | 0.01±0.01 | 0.01±0.01 | 0.01±0.002 |
| 1667 | Ribose, tetra TMS |  | 0.01±0.002 | 0.01±0.002 | 0.01±0.005 | 0.02±0.02 | 0.01±0.003 | 0.01±0.001 |
| 1683 | Arabinose, tetra-O-TMS |  | 0.01±0.001 | 0.01±0.001 | 0.01±0.004 | 0.02±0.02 | 0.01±0.004 | 0.01±0.001 |
| 1690 | Rhamnose, tetra TMS |  | 0.02±0.002 | 0.03±0.002 | 0.03±0.03 | 0.07±0.06 | 0.09±0.01 | 0.01±0.0004 |
| 1731 | Rhamnose, tetra TMS |  | 0.04±0.0003 | 0.05±0.002 | 0.06±0.06 | 0.13±0.11 | 0.16±0.02 | 0.02±0.001 |
| 1826 | Mannose, penta TMS |  | 0.06±0.01 | 0.08±0.01 | 0.03±0.01 | 0.02±0.01 | 0.03±0.01 | 0.004±0.001 |
| 1829 | Fructose, penta TMS |  | 0.01±0.01 | 0.07±0.09 | 0.01±0.02 | 0.002±0.0004 | 0.01±0.004 | 0.002±0.001 |
| 1837 | Galactofuranose, penta TMS |  | 0.30±0.03 | 0.24±0.06 | 0.28±0.08 | 0.30±0.07 | 0.20±0.02 | 0.06±0.01 |
| 1856 | 2-deoxy-galactopyranose, tetra TMS |  | 0.23±0.01 | 0.10±0.003 | 0.11±0.07 | 0.07±0.07 | 0.46±0.06 | 0.03±0.01 |
| 1881 | Galactopyranose, penta TMS ether |  | 0.21±0.01 | 0.16±0.01 | 0.25±0.14 | 0.40±0.27 | 0.17±0.01 | 0.50±0.03 |
| 1915 | Mannose, penta TMS |  | 0.58±0.03 | 1.07±0.58 | 0.20±0.11 | 0.28±0.20 | 0.17±0.02 | 0.36±0.06 |
| 1926 | Talopyranose, penta TMS ether |  | 0.51±0.07 | 0.44±0.02 | 0.58±0.36 | 0.87±0.61 | 0.37±0.02 | 1.01±0.06 |
| 1999 | D-Glucopyranose, TMS |  | 0.70±0.0004 | 1.14±0.69 | 0.23±0.13 | 0.33±0.20 | 0.17±0.01 | 0.49±0.05 |
| 2021 | Galacto furanoside, ethyl 2,3,5,6-tetra-O-TMS |  | 0.01±0.003 | 0.002±0.001 | 0.002±0.001 | 0.004±0.002 | 0.002±0.0004 | 0.003±0.001 |
| 2027 | Gluconic acid, hexa TMS |  | 0.17±0.001 | 0.05±0.003 | 0.05±0.02 | 0.04±0.02 | 0.11±0.01 | 0.52±0.02 |
| 2661 | Maltose, octa TMS ether |  | 0.01±0.001 | 0.01±0.003 | 0.01±0.004 | 0.005±0.001 | 0.01±0.001 | 0.01±0.002 |
| 2691 | D-Lactose, octa TMS ether* |  | 37.94±2.56 | 43.96±3.69 | 41.37±3.50 | 38.73±4.71 | 48.44±2.31 | 43.24±0.52 |
| 2799 | Unknown sugar |  | 1.70±0.12 | 1.84±0.15 | 1.75±0.16 | 1.67±0.27 | 2.21±0.17 | 2.14±0.06 |
| 2803 | D-Lactose, octa TMS ether |  | 18.33±1.53 | 18.01±2.33 | 18.86±1.65 | 20.21±3.10 | 21.81±1.88 | 25.15±0.39 |
| 2876 | 3-α-Mannobiose, octa TMS ether |  | 0.03±0.001 | 0.03±0.0002 | 0.03±0.002 | 0.03±0.002 | 0.03±0.01 | 0.03±0.004 |
| 2937 | Galactinol, nona TMS ether |  | 0.06±0.001 | 0.14±0.01 | 0.06±0.01 | 0.07±0.04 | 0.13±0.05 | 0.54±0.13 |
|  | **Total sugars** | | **60.94±1.25** | **67.47±2.69** | **63.95±4.42** | **63.29±6.44** | **74.62±0.43** | **74.16±0.44** |
| 1983 | Mannonic acid, 1,5-lactone, tetra TMS | Sugar acid | 0.005±0.0001 | 0.01±0.0004 | 0.004±0.001 | 0.01±0.002 | 0.01±0.001 | 0.005±0.001 |
| 1992 | Glucuronic acid, penta TMS |  | 0.01±0.001 | 0.01±0.002 | 0.01±0.004 | 0.01±0.004 | 0.01±0.005 | 0.01±0.002 |
|  | **Total sugar acids** | | **0.01±0.001** | **0.02±0.002** | **0.02±0.005** | **0.02±0.01** | **0.02±0.01** | **0.01±0.003** |
| 1517 | Erythritol, tetra TMS | Sugar alcohol | 0.05±0.002 | 0.05±0.003 | 0.04±0.003 | 0.05±0.001 | 0.06±0.003 | 0.06±0.003 |
| 1738 | Arabinitol, penta-O-TMS |  | 0.02±0.0003 | 0.02±0.0003 | 0.02±0.01 | 0.02±0.02 | 0.01±0.002 | 0.01±0.002 |
| 1743 | Ribitol, penta TMS |  | 0.003±0.001 | 0.003±0.0005 | 0.002±0.001 | 0.003±0.002 | 0.002±0.0004 | 0.01±0.001 |
| 1849 | 1,5-Anhydro-sorbitol, tetra TMS ether |  | 0.48±0.02 | 0.16±0.01 | 0.33±0.21 | 0.22±0.18 | 0.09±0.02 | 0.05±0.01 |
| 1961 | Mannitol, hexa TMS |  | 0.02±0.001 | 0.02±0.001 | 0.02±0.03 | 0.02±0.03 | 0.05±0.004 | 0.07±0.003 |
| 2118 | Myoinositol, hexa TMS |  | 0.59±0.04 | 1.57±0.04 | 0.44±0.18 | 0.57±0.32 | 1.29±0.003 | 2.20±0.09 |
|  | **Total sugar alcohols** | | **1.15±0.06** | **1.82±0.03** | **0.86±0.16** | **0.89±0.33** | **1.50±0.02** | **2.40±0.09** |
| 1295 | Nicotinic acid TMS (vitamin B3 or niacin) | Vitamin | 0.001±0.001 | 0.001±0.0001 | 0.001±0.0003 | 0.002±0.001 | 0.001±0.0001 | 0.001±0.001 |
| 1778 | Ribonic acid, penta TMS (folic acid or vitamin B9) |  | 0.20±0.001 | 0.32±0.03 | 0.19±0.08 | 0.28±0.22 | 0.18±0.02 | 0.64±0.03 |
| 1970 | Ascorbic acid, tetra TMS (vitamin C) |  | 0.03±0.01 | 0.07±0.01 | 0.01±0.001 | 0.02±0.01 | 0.03±0.02 | 0.13±0.01 |
|  | **Total vitamins** | | **0.23±0.01** | **0.40±0.03** | **0.20±0.08** | **0.29±0.23** | **0.21±0.01** | **0.77±0.04** |
